# CD47 promotes ovarian cancer progression by inhibiting macrophage phagocytosis

**DOI:** 10.18632/oncotarget.16547

**Published:** 2017-03-24

**Authors:** Ran Liu, Huiting Wei, Peng Gao, Hu Yu, Ke Wang, Zheng Fu, Baohui Ju, Meng Zhao, Shangwen Dong, Zhijun Li, Yifeng He, Yuting Huang, Zhi Yao

**Affiliations:** ^1^ Tianjin Key Laboratory for Modern Drug Delivery & High-Efficiency, School of Pharmaceutical Science and Technology, Tianjin University, Tianjin, 300072, China; ^2^ Department of Immunology, Tianjin Key Laboratory of Cellular and Molecular Immunology, Key Laboratory of Educational Ministry of China, School of Basic Medical Sciences, Tianjin Medical University, Tianjin, 300071, China; ^3^ University of the District of Columbia, Washington D.C., 20008, United States; ^4^ Tianjin Medical University Cancer Institute and Hospital, National Clinical Research Center of Cancer, Key Laboratory of Cancer Prevention and Therapy, Tianjin, 300040, China; ^5^ Second Hospital of Tianjin Medical University, Tianjin, 300211, China; ^6^ Tianjin Medical University General Hospital, Tianjin, 300052, China; ^7^ Department of Gynecology and Obstetrics, Renji Hospital, Shanghai, 200127, China

**Keywords:** ovarian cancer, CD47, macrophage, phagocytosis, monoclonal antibody

## Abstract

Targeting CD47 efficiently enhances macrophage phagocytosis in both physiological and pathological conditions. Anti-CD47 antibodies have been shown to inhibit the progression of several types of cancer. However, the mechanism of anti-CD47 monoclonal antibody (mAb) treatment remains controversial. In this study, we confirmed that CD47 protein is highly expressed in ovarian cancer, and is correlated with poor clinical characteristics and prognosis. CD47 knockdown in the ovarian cancer cell line, SK-OV-3, promoted phagocytosis by macrophages *in vitro* and inhibited tumor growth *in vivo*. These data combined suggest that CD47 inhibition is a potential strategy for cancer treatment. Using an anti-CD47 mAb, we found that CD47 inhibition in both SK-OV-3 cells and primary cancer cells was able to recapitulate our knockdown results and led to an increase in the number of infiltrating macrophages. In addition, the CD133^+^ tumor initiating cells expressed a high level of CD47, and anti-CD47 mAb treatment was able to trigger the phagocytosis of this cell population. In conclusion, our results indicate that CD47 inhibits macrophage phagocytosis of ovarian cancer cells, and down-regulation of CD47 or inhibiting CD47 by mAb was able to reverse the negative effect. Thus, CD47 antibody therapy may be a promising strategy to treat ovarian cancer.

## INTRODUCTION

Ovarian cancer is the most lethal gynecological malignancy, and ranks fifth in female cancer-related deaths. Ovarian cancer accounts for up to 3% of malignant diseases in women, and the mortality of this disease accounts for up to 5% [1]. Unfortunately, ovarian cancer is often asymptomatic at early stages. Consequently, approximately 80% of patients are already at the advanced stages of the disease when diagnosed [2]. Mortality is highly related to metastasis-related complications, such as cachexia and multiple organ dysfunction syndromes. Therefore, the elucidation of the mechanisms underlying ovarian cancer metastasis and the strategies to prevent this process are needed in order to improve treatment of ovarian cancer.

In contrast to other malignant epithelial diseases, epithelial ovarian cancer most frequently disseminates by implantation within the peritoneal cavity due to the unique anatomical characteristics of the ovary [3]. Once invaded, the epithelial ovarian cancer cells migrate along the peritoneal cavity and can easily seed within this space. Macrophages are an essential member of the innate immune system, and play a critical role in anti-tumor immune response [4]. Although, macrophages are abundant in the peritoneal cavity [5], it remains unclear how ovarian cancer cells are able to escape the anti-tumor immune-surveillance of the macrophages.

CD47 also known as integrin-associated protein (IAP) is a membrane protein of the immunoglobulin superfamily. It participates in integrin and cadherin signaling by interacting with thrombospondin-1 (TSP-1) and signal regulatory protein alpha (SIRPα) [6]. This interaction negatively regulates macrophage phagocytosis through inhibitory signaling. This mechanism is involved in several physiological processes including red blood cell clearance [7] and hematopoietic stem cell homing [8]. Under pathological conditions, CD47 expression on the cancer cell membrane suppresses the phagocytic activity of immune cells and is associated with poor prognosis of bladder cancer, leukemia, non-Hodgkin's lymphoma, and breast cancer [9]. These data suggest that CD47 inhibition may trigger the innate immune system, particularly macrophage phagocytosis, and further activate the adaptive immune system in clearing tumor cells [10].

In several pre-clinical studies, CD47 monoclonal antibody (mAb) treatment has shown impressive anti-tumor effects in xenograft cancer models. However, there is no consensus on the contribution of the CD47-SIRPα axis in anti-CD47 mAb therapy. In this study, we confirmed the clinical significance of CD47 in ovarian cancer, showed the presence of CD47 dependency in anti-CD47 therapy, and investigated the possibility of using anti-CD47 mAb as a treatment paradigm.

## RESULTS

### CD47 is highly expressed in ovarian cancer and correlates with poor clinical outcome

**Figure 1:**
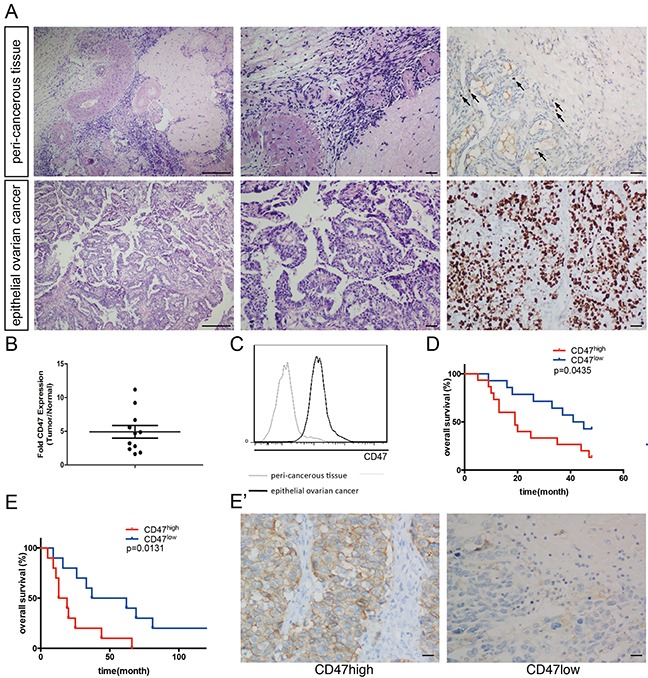
CD47 is highly expressed in ovarian cancer and correlates with poor clinical outcome **(A)** Representative pathology of patients. Left: ×40 magnification H&E staining (scale bar = 250 μm); middle: ×100 magnification H&E staining (scale bar = 50 μm); right: ×100 magnification IHC staining of Ki-67 (scale bar = 50 μm). **(B)** CD47 expression in the epithelial cancer tissues and peri-cancerous tissues in a representative patient. **(C)** Relative CD47 expression in epithelial ovarian cancer tissues normalized to morphologically normal peri-cancerous tissues, measured by MFI fold change using flow-cytometry. **(D)** Kaplan-Meier survival curve of CD47^high^ (n = 15) and CD47^low^ (n = 14) patients in the high-grade serous ovarian cancer cohort (Log-rank test, *p < 0.05). **(E)** Ten-year survival curve of CD47^high^ (n = 10) and CD47^low^ (n = 10) patients in the high-grade serous ovarian cancer (Log-rank test, *p < 0.05). **(E')** Representative IHC staining of CD47 in the high-grade serous ovarian cancer cohort (scale bar = 50 μm).

CD47 has been reported to be an ovarian cancer marker [11]. CD47 expression is elevated in ovarian cancer and indicates poor prognosis [9]. In the current study, tumor tissues and morphologically normal peri-cancerous tissues were obtained by a pathologist and confirmed by hematoxylin and eosin (H&E) staining and immunohistochemistry (IHC) staining of Ki-67 (Figure 1A). CD47 expression was quantified by relative mean fluorescence intensity determined by flow-cytometry. In all 11 patients, CD47 expression levels were significantly higher in the tumor tissues than the normal tissues with an approximate 4.9-fold increase in intensity (Figure 1B and 1C).

To further investigate the clinical significance of CD47 expression, we subdivided another cohort of 93 ovarian cancer patients based on their CD47 expression levels. Levels of the CD47 expression from the samples are quantified by measuring the mean fluorescent intensity by flow cytometry. The 40 patients with the highest CD47 expression were categorized as CD47^high^, and the 40 patients with the lowest CD47 levels as CD47^low^. The 13 patients with medium CD47 expression levels were excluded in the analyze. The Patients' clinical characteristics are summarized in Table 1. In the CD47^high^ group, 39 out of 40 patients (97.5%) were in International Federation of Gynecology and Obstetrics (FIGO) stage III-IV. By contrast, stage III-IV patients only constituted 77.5% of the CD47^low^ group (χ^2^=7.31, p < 0.01). Patients with poorly differentiated or undifferentiated tumors made up 47.5% of the CD47^low^ group, but these patients represented a significantly higher proportion of the CD47^high^ group (67.5%, χ^2^=6.37, p < 0.05). This observation indicates a linkage between CD47 expression and poor clinical characteristics.

**Table 1:** Distribution of ovarian cancer patients by tumor characteristics

Group (n)		CD47^high^ 40	CD47^low^ 40
Clinical Parameters			
Histology Type			
	Serous	17	16
	Mucinous	13	17
	Endometrioid	8	7
	Clear cell	2	0
FIGO Stage	χ2=7.31 p<0.01		
	Stage I-II	1	9
	Stage III-IV	39	31
Pathological Grading	χ2=6.37 p<0.05		
	Well/Moderately differentiated	10	21
	Poorly differentiated/Undifferentiated	30	19

To exclude the impact of pathology grade and clinical FIGO stages on CD47 expression in patient survival, we assessed 29 FIGO stage III-IV patients with poorly differentiated serous adenocarcinoma for 50 months. The Kaplan-Meier survival curve showed poorer overall survival for the 15 CD47^high^ patients (Figure 1D). Since the 10-year survival rate has become a well-accepted assessment of high-grade serous ovarian cancer, we performed IHC staining using anti-CD47 antibodies on another cohort of patients who were diagnosed with high-grade serous ovarian cancer 10 years ago, and found that a poorer prognosis was also observed in CD47^high^ patients compared to CD47^low^ patients (Figure 1E and 1E').

### CD47 knockdown promotes *in vitro* phagocytosis and inhibits *in vivo* tumor formation in ovarian cancer cell line SK-OV-3

**Figure 2:**
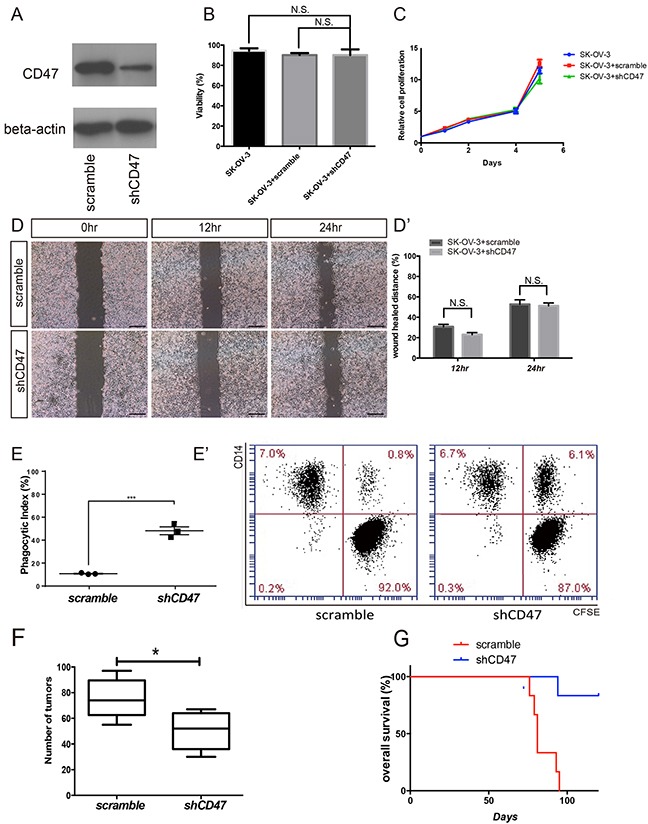
CD47 knockdown promotes *in vitro* phagocytosis and inhibits *in vivo* tumor formation in the ovarian cancer cell line SK-OV-3 **(A)** Expression of CD47 in SK-OV-3 cells transfected by shCD47 or scramble shRNA was examined by Western blot analysis. Full-length blots are presented in Supplementary Figure 2. **(B)** Knocking down CD47 expression by RNAi in SK-OV-3 cell line did not affect cell viability. **(C)** MTT assays showed no significant change in proliferation after SK-OV-3 cells were transfected by shCD47 or scramble shRNA. **(D)** Knocking down CD47 expression had no significant impact on cell migration analyzed by wound-healing assays (scale bar = 100 μm). **(D')** Wound distance percentage quantification. **(E)** The phagocytic index increased in the CD47 knockdown SK-OV-3 cells. (Unpaired Student's-t-test, two-tailed, ***p < 0.01), **(E')** Representative flow cytometry results are shown. **(F)** Quantification of tumor numbers in mice xenografted with SK-OV-3 cells transfected with shCD47 or scramble shRNA (Unpaired Student's-t test, two-tailed, *p < 0.05). **(G)** Kaplan-Meier plots show better overall survival in mice xenografted with CD47 knockdown SK-OV-3 cells (N=6, Log-rank test, **p < 0.01).

In the canonical CD47 pathway, the CD47-SIRPα interaction initiates a “don't eat me” signal. Activation of this signaling cascade is responsible for the escape of these cells from immune-surveillance. A previous study showed that an anti-CD47 mAb treatment significantly reduced the tumor load in various types of malignant diseases [9]. Ovarian cancer has a unique metastatic process in which the invasive tumor cells float directly into the peritoneal cavity, thereby making the interaction between cancer cells and peritoneal macrophages critical in metastasis. It remains unclear whether the elevated phagocytosis by CD47 mAb treatment is a nonspecific antibody dependent effect or a result of disrupting CD47 intrinsic functions. Thus, we suppressed CD47 expression in the SK-OV-3 ovarian cancer cell line using short-hairpin RNAs (shRNA) that target CD47 transcripts. The CD47 protein levels were significantly reduced in cells transduced with shCD47 when compared with cells transduced with a control scramble shRNA (Figure 2A). Cells treated with shCD47 had no significant changes in tumor cell proliferation, viability, or migration (Figure 2B-2D). To test whether down-regulation of CD47 promotes macrophage phagocytosis, we determined the phagocytic index by flow-cytometry. The phagocytic index, defined as the percentage of the macrophages that engulfed tumor cells out of total macrophages, was quantified using flow-cytometry. Scramble control and CD47 knockdown SK-OV-3 cells were incubated with human macrophages derived from the monocytic cell line, THP-1. In scramble shRNA transduced SK-OV-3 cells, the baseline phagocytic index was as low as 10%, whereas the phagocytic index increased to more than 40% in CD47-shRNA transduced SK-OV-3 cells (Figure 2E and 2E'). To evaluate whether this effect can be reproduced *in vivo*, CD47 knockdown SK-OV-3 cells and scramble shRNA control cells were xenografted to BALB/c nude mice via intra-peritoneal injection to mimic intra-peritoneal metastasis. As expected, all animals developed tumors within 4 weeks of the xenograft, and were primarily found on the mesentery. However, we observed fewer xenograft tumors in the CD47 knockdown group than in the control group (Figure 2F, Supplementary Table 1). The Kaplan-Meier survival curve also showed significantly improved survival in mice xenografted with CD47 knockdown cells than in controls (Figure 2G).

### The CD47 monoclonal antibody promotes phagocytosis *in vitro*, inhibits tumor formation, and enhances macrophage infiltration *in vivo*

**Figure 3:**
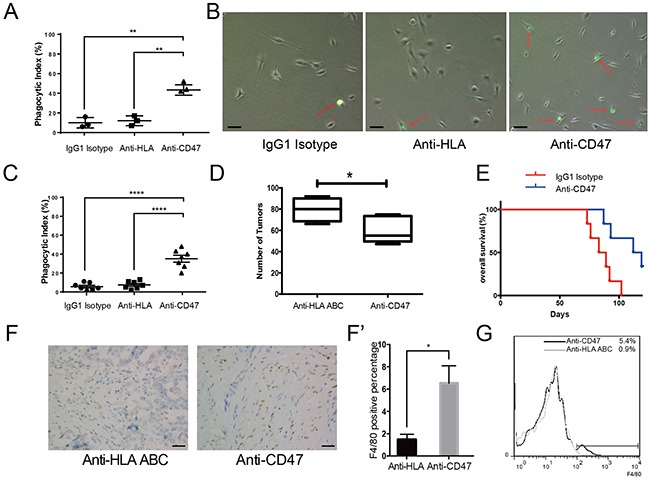
The CD47 monoclonal antibody promotes phagocytosis *in vitro*, inhibits tumor formation and enhances macrophage infiltration *in vivo* **(A)** CD47 mAb promoted phagocytosis of SK-OV-3 cells *in vitro*. **(B)** Representative bright field microscopy of phagocytosis assays. (Scale bar = 20 μm) **(C)** Anti-CD47 mAb increased the phagocytic index of primary ovarian cancer cells obtained from 7 patients. **(D)** Quantification of tumor numbers in anti-CD47 mAb treated mice and anti-HLA antibody treated control mice (Unpaired Student's-t test, two-tailed, *p < 0.05). **(E)** Kaplan-Meier survival plot of anti-CD47 mAb treated SK-OV-3 xenograft nude mice shows prolonged survival (n = 6, Log-rank test, **p < 0.01). **(F)** IHC staining of F4/80 in the tumors from SK-OV-3 cells xenograft nude mice treated with anti-CD47 mAb or anti-HLA antibody. (Scale bar = 50 μm). **(F')** Quantification of the percentage of F4/80 positive cells in the tumors based on IHC staining. **(G)** Representative flow-cytometry result shows more infiltrating macrophages in the tumor from anti-CD47 mAb treated SK-OV-3 xenograft nude mice.

Although mAb and gene editing treatment paradigms have been intensively investigated for cancer treatment, the underlying safety issues associated with gene editing can limit its clinical applications. Monoclonal antibody treatment, however, is considered safer and more clinically applicable in ovarian cancer therapies. Due to the unique anatomical characteristics, ovarian cancer is prone to implant within the peritoneal cavity. Unfortunately, approximately 80% of patients are asymptomatic and are already in the advanced stages of disease progression upon diagnosis. However, interrupting the early seeding of the floating cancer cells for the other rest 20% of the patient population may be critical in improving the clinical outcomes. To test whether mAb treatment would be a viable therapeutic approach, we designed *in vitro* phagocytosis assays and *in vivo* experiments similar to the shRNA knockdown experiments and evaluated the efficacy of an anti-CD47 mAb as a therapeutic in ovarian cancer, especially in preventing the early seeding events. CFSE labeled SK-OV-3 cells were mixed with CD47 mAb, anti-human leukocyte antigen (HLA) ABC antibody, or IgG1 isotype. These cells were then co-cultured with THP-1-derived macrophages to measure the phagocytic index. CD47 inhibition by mAb significantly increased the phagocytic index compared to controls. Flow-cytometry showed that 46% of the macrophages engulfed cancer cells after the CD47 signal was blocked, which was much higher than that of the anti-HLA antibody group or the IgG1 isotype group (Figure 3A and 3B). We also performed the same experiment on ascites tumor cells isolated from 7 epithelial ovarian cancer patients. In all 7 cases, the phagocytic index increased significantly after CD47 blockade (Figure 3C).

To determine whether CD47 mAb treatment would also work *in vivo*, SK-OV-3 cells were mixed with the anti-CD47 mAb prior to xenografting, then the same antibody was administered to the animals intermittently weekly after the xenograft. In the anti-CD47 mAb treated xenograft group, the number of tumors was significantly lower than that of the control group (Figure 3D, Supplementary Table 2). Additionally, the anti-CD47 mAb treatment group survived significantly longer than the anti-HLA control group (Figure 3E).

Macrophages are important tumor infiltrating cells [12]. To investigate the macrophage infiltration in xenograft tumors, individual tumors were resected and examined for the presence of macrophages by both IHC staining (Figure 3F) and flow-cytometry (Figure 3G) using the murine macrophage marker F4/80. Consistent with a previous report in a human liver cancer xenograft model [13], we observed that approximately 6% of the total tumor mass was infiltrated by macrophages when CD47 was blocked by the antibody, whereas only less than 1% was infiltrated in control (Figure 3F').

### CD47 expression is elevated in the ovarian cancer initiating cells

**Figure 4:**
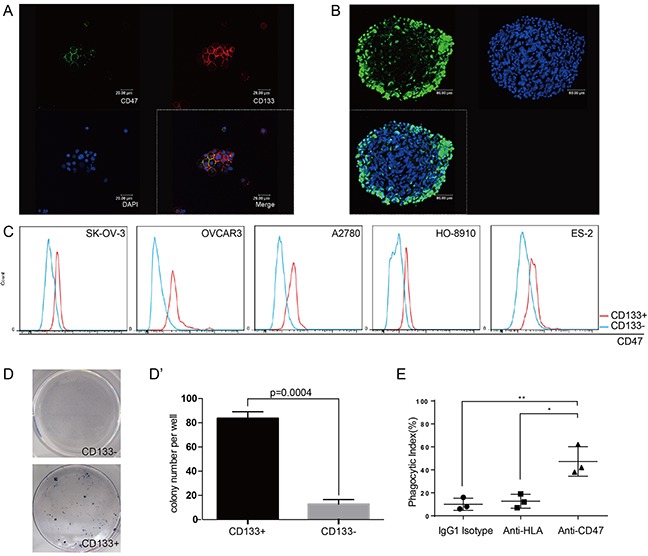
CD47 expression is elevated in the ovarian cancer stem cell-like population **(A)** Tumor cells obtained from patient ascites samples were used for sphere forming assays. CD47 and CD133 expression on the spheres was analyzed by immunofluorescence staining (scale bar = 20 μm). **(B)** Ovarian cancer sphere cells showed aldehyde dehydrogenase (scale bar = 20 μm). **(C)** The CD47 expression levels in CD133^+^ and CD133^−^ sub-populations was measured by mean fluorescence intensity in 5 epithelial ovarian cancer cell lines. **(D)** Representative colony formation assay for HO-8910 CD133^+^ and CD133^−^ sub-populations. **(D')** The number of colonies was quantified and plotted (Unpaired Student's-t-test, two-tailed, **p < 0.005). **(E)** The anti-CD47 mAb promoted phagocytosis of the CD133^+^ sub-population of cells isolated from the HO-8910 cell line by macrophages.

It is well accepted that CD133 expression defines a tumor initiating cell (TIC) population in primary human ovarian cancer [14], and CD133 is often an indicator of a resistance phenotype [15]. After the initial success of therapeutic treatment, these resistant cells persist and often cause the development of cancers or the secondary tumors leading to relapse. Primary ovarian cancer cells isolated from patient ascites samples were cultured under stem cell culture condition, and formed spheres. These primary ovarian cancer spheres were found to express both CD47 and the TICs marker CD133 (Figure 4A). These spheres also showed high levels of aldehyde dehydrogenase (ALDH) activity, which has been reported to play a role in maintaining stem cell-like properties [16, 17] (Figure 4B). These data led us to use the CD133 label as our criteria to define ovarian cancer initiating cells.

We first examined the CD47 expression levels in the CD133^+^ and CD133^−^ sub-populations in five different epithelial ovarian cancer cell lines. The CD133^+^ sub-population showed higher mean fluorescence intensities (MFI) of CD47 compared to the CD133^−^ sub-population (Figure 4C). Colony-forming assays were then performed on sorted sub-populations. The ability of a sorted cell to form a colony indicated the tumorigenic capability of these single cells. There were significantly more colony-forming cells in the CD133^+^ sub-population compared to CD133^−^ sub-population (Figure 4D and 4D'), indicating that the CD133^+^ sub-population is highly enriched for the tumor initiating cells. Since the high percentage of CD133^+^ cells found in the HO-8910 cell line would avoid prolonged cell sorting (Supplementary Figure 1), we isolated CD133^+^ cells from this cell line and performed phagocytosis assays. The phagocytic index of the anti-CD47 mAb group was significantly higher compared with that of the anti-HLA antibody group or the IgG1 isotype group (Figure 4E). These results suggested that anti-CD47 mAb can efficiently trigger the phagocytosis of CD113+ cells by macrophages, indicating that anti-CD47 that anti-CD47 mAb can be used as a new therapeutic strategy for clinical cancer treatments in the future.

## DISCUSSION

In agreement with a previous study [9], we found that CD47 was highly expressed in ovarian cancer and was associated with poor clinical characteristics and prognosis. In addition, CD47 knockdown using shRNA in the SK-OV-3 ovarian cancer cell line promoted *in vitro* macrophage phagocytosis and inhibited tumor initiation *in vivo*, suggesting that such an anti-tumor effect is CD47 dependent. Since inhibitory antibody administration is more clinically applicable, we further blocked the CD47 pathway in both SK-OV-3 cells and primary patient cancer cells using the anti-CD47 mAb, BRIC126. Similar to shRNA knockdown, anti-CD47 mAb increased *in vitro* macrophage phagocytosis, decreased tumor initiation *in vivo*, and most importantly, promoted macrophage infiltration into xenograft tumors. Additionally, high CD47 expression was detected in the CD133^+^ TIC population, and anti-CD47 mAb triggered the phagocytosis of these cells, which are relatively resistant to the current treatment paradigms. Taken altogether, our results indicated that CD47 promotes ovarian cancer progression by inhibiting macrophage phagocytosis, and knockdown of CD47 or anti-CD47 mAb treatment can reverse this effect. Thus, anti-CD47 therapy may provide a complementary way to target the TICs population to prevent metastasis and cancer recurrence.

This study employed a suitable mouse model for xenograft experiments. There is no recognized mouse ovarian cancer cell line. Therefore *in vivo* studies on ovarian cancer often use human ovarian cancer cell line xenograft mouse models. Xenografting human cancer cell line into murine model often results in rejection. Therefore, immunocompromised mice are widely being used in oncology research. Compared to mice of the NOD background, BALB/c mice have intact phagocytic functions in macrophages [18]. The scope of our study is focused on the anti-tumor function of macrophage. As a result, after careful evaluation, we chose the BALB/c nude mice, an immunocompromised model that has an intact macrophage response.

There has been a controversy on the mechanism of anti-tumor effects induced by anti-CD47 mAbs [18]. Some people argue that the Fc fragment might be participating in the anti-tumor effect of anti-CD47 mAb. Fc-dependent effects such as opsonization and ADCC should be taken into consideration. Kipp Weiskopf *et al*., showed that antagonizing the CD47-SIRPα interaction had minimal anti-tumor effects *in vivo* using Fab fragments without Fc [19]. To examine the potential role of the Fc fragment, anti-HLA control antibody [20] control antibody was used in the current study. Ovarian cancer cells express relatively high levels of HLA. The use of an anti-HLA control antibody can contribute sufficient Fc fragments but no increase in phagocytosis was observed, indicating such a therapeutic effect is independent of the Fc fragment. These results are further supported by studies showing that CD47 blocking mAb, B6H12, improves phagocytosis while a non-blocking mAb 2D3 [9] and anti-CD45 antibody [21] do not, despite the presence of Fc. F(ab)'2 fragment of anti-CD47 antibody does the same in the absence of Fc [22]. The controversy over Fc may be of particular interest in pharmaceutics. Two different anti-CD47 antibodies currently in clinical trials have adopted different strategies [18]. TTI-621 engages IgG1, while Hu5F9-G4 uses the Fc of IgG4 to minimize ADCC and CDC [23]. The two strategies may result in different balance between safety and efficacy.

Ovarian cancer has the highest mortality rates of all gynecological malignancies. Ovarian cancer is typically asymptomatic in the early stages, but can easily disseminate and metastasize [1]. Nearly 80% of the patients are diagnosed after the disease has become symptomatic and has progressed to a late stage. Despite high response rates to platinum-based chemotherapeutics, many patients will experience recurrence within two years. For those patients with recurrent ovarian cancer, many attempts have been made to address the difficult “watchful waiting” periods between the platinum-based chemotherapy cycles. Anti-CD47 mAb gives a promising option for treatment, showing promising therapeutic benefits with few safety issues. In most solid tumors, mAbs are often given via intra-venous injection. However, intra-venous application of anti-CD47 antibody may have potential risks. High levels of CD47 expression protects red blood cells (RBCs) from intra-splenic macrophage phagocytosis [7]. Hence, loss of CD47 on RBCs may cause hemolysis and anemia [24]. In a preclinical study on acute myelogenous leukaemia (AML), anti-CD47 mAb was delivered via intra-venous [23]. In this study, anemia is only transient and can be further mitigated with a specific dose of erythropoietin (EPO) [23]. While EPO can overcome anemia, the impact of hemolysis, such as hyperbilirubinemia, still needs further evaluation. In the case of ovarian cancer, its anatomic and structural characteristics make it easy to disseminate and metastasize within the peritoneal cavity, such that intra-peritoneal delivery of the medication may be a viable option. It has already been reported that in nude mice with human-derived ovarian tumors, tumor uptake of specific antibody via intra-peritoneal injection was significantly higher than that following intra-venous antibody delivery [25].

Unfortunately, these malignant tumors do not have clear margins for satisfying surgical removal, resulting in residual tumor cells being left behind. This often leads to a risk of iatrogenic dissemination, and eventually, ovarian cancer recurrence. Once these tumor-forming cells have invaded, the tumor cells will float, spread, and seed within the peritoneal cavity. Eliminating this small load of tumor cells and preventing them from seeding is critical in reducing the risk of recurrence. Our results suggested that applying anti-CD47 mAb during the “watchful waiting” period is expected to eliminate these tumor seeding cells and further benefit the patients' long term prognosis.

In 2005, Bapat *et al*. reported ovarian cancer stem cells (CSCs) for the first time [26]. Ovarian CSCs behave like “seeds” and play a crucial role in tumor initiation, growth, invasion, metastasis, and recurrence [27]. Although the concept of cancer stem cells is still controversial, it is widely accepted that clonal evolution exists in cancer initiation and progression [28]. We analyzed the CD47 expression profile in the tumor initiating cells of ovarian cancer to determine its role in immune escape. While chemotherapies and radiotherapies are able to target the most proliferative cell populations, enabling them to kill cancer cells effectively [29], this feature can often lead to many side effects by damaging labile cells such as bone marrow cells. Also, the existing treatments mostly target the highly proliferative cancer cells, leaving the relatively quiescent TICs intact. While these cells may show different responses to chemotherapies, cancer cells and the TICs both express high levels of the ‘don't eat me’ signal CD47 to prevent the macrophages from engulfing and eliminating them. Our current study shows that inhibition using anti-CD47 mAb, which enables macrophages to eliminate tumor cells, could also induce robust phagocytosis of the TICs. This finding is novel in the context of the fact that TICs are resistant to most of the current treatment paradigms, which highlights the potential therapeutic applications of this anti-CD47 mAb in reducing tumor recurrence. While a similar strategy has been explored in managing AML [21], no such studies have been done in the management of gynecological malignant diseases.

Our results support an intrinsic role of CD47 in ovarian cancer progression and immune evasion. While CD133^+^ cells are often resistant to chemotherapies and radiotherapies, anti-CD47 mAb treatment showed significant efficacy to this cell population. Adding anti-CD47 mAb therapy as a supplement to the standard therapeutic schemes may prevent ovarian cancer metastasis and recurrence.

## MATERIALS AND METHODS

### Clinical data collection

All the protocols including clinical data and sample collection were approved by the supervising board of Tianjin Medical University Cancer Hospital and Institute. The procedures were performed in accordance with the approved guidelines. All patients met the enrollment criteria and provided signed informed consent. The collected information included the expression levels of tumor markers, medical imaging results, surgery procedures, pathological classifications, sensitivity to chemotherapy, recurrence, progression free survival (PFS), and overall survival.

### Flow-cytometry

In *in vitro* experiments, single cell suspension was obtained by trypsin digestion. Ovarian cancer specimens were digested by collagenase to obtain single cells. Then, 1×10^6^ cells were washed, and re-suspended in 100 μL phosphate-buffered saline (PBS) and stained with PE-conjugated anti-human CD133 (AC133, MiltenyiBiotec, 130-080-801), PE-conjugated mouse IgG1 isotype (IS5-21F5, MiltenyiBiotec, 130-092-212), APC-conjugated anti-human CD47 (B6H12, eBioscience, 17-0479), APC-conjugated anti-human CD14 (63D3, BioLegend, 367118) and APC-conjugated mouse IgG1κ isotype control (MOPC-21, BioLegend, 400122) antibodies. All samples were incubated for 30 minutes at 4°C, washed twice with 2% FBS in PBS, and preserved by addition of 0.6 mL 0.2% PFA. CFSE (eBioscience, 65-0850) labeling and aldehyde dehydrogenase activity assays (ALDEFLUOR™ Kit, Stemcell Technologies, 01700) were performed according to the manufacturers' protocols. Flow-cytometry data were acquired using a FACS C6 Flow Cytometer (Accuri Cytometer). At least 30,000 events were collected for each analysis. All data were further analyzed by FlowJo software (Tree Star). The relative MFI of CD47 expression was calculated by dividing the experimental sample MFI by the isotype control MFI. The fold change in CD47 expression was calculated as equation (1):

$$\text{CD}47\text{ exression fold change}=\frac{\text{cancer CD}47\text{ MFI}/\text{cancer isotype MFI}}{\text{pericancerous CD}47\text{ MFI}/\text{pericancerous isotype MFI}}$$ (1)

### Cell culture and macrophage differentiation

The human epithelial ovarian cancer cells lines (SK-OV-3, A2780, OVCAR3 and HO-8910), human ovarian clear cell carcinoma (ES-2) and a human monocytic cell line (THP-1) were purchased from the Type Culture Collection of Chinese Academy of Sciences and maintained according to their recommendations. SK-OV-3 and ES-2 cells were cultured using McCoy's 5a medium (ThermoFisher Scientific, 16600082) supplemented with 1% HEPES (ThermoFisher Scientific, 15630080), 1% penicillin-streptomycin-glutamine (ThermoFisher Scientific, 10378016) and fetal bovine serum to a final concentration of 10%. OVCAR3 cells were cultured using RPMI-1640 medium (ThermoFisher Scientific, 11875135) supplemented with 1% HEPES, 1% penicillin-streptomycin-glutamine and fetal bovine serum to a final concentration of 20%. A2780, HO-8910 andTHP-1 cells were cultured using RPMI-1640 medium supplemented with 1% HEPES, 1% penicillin-streptomycin-glutamine and fetal bovine serum to a final concentration of 10%. THP-1 cells were differentiated into macrophages by addition of PMA (50 ng/mL, Sigma-Aldrich) and overnight incubation. This was followed by 2 washes with THP-1 cell culturing medium, after which the cells were cultured for over 48h post differentiation prior to other experiments.

### Phagocytosis assay

Cancer cells were digested into single cell suspensions, and then labeled with CFSE (eBioscience, 65-0850) following the manufacturer's protocol. Then, they were exposed to 20 μg/mL purified mouse anti-human CD47 antibody (BRIC 126, AbDSerotec, MCA911). Controls were exposed to the same doses of mouse anti-human IgG2b and mouse anti-human HLA ABC antibody (AbDSerotec). CFSE-labeled cancer cells were co-cultured with macrophage cells for 2h. The phagocytic index was calculated by dividing the number of macrophages engulfing tumor cells by the total number of macrophages.

### RNAi of CD47 in SK-OV-3 cell line

The CD47-shRNA and control sequences were obtained from Shanghai GenePharma Co., Ltd. The sequences are as follows: CD47-shRNA (5′-CGTCACAGGCAGGACCCACTGCCCA-3′), scramble-shRNA (5′-CGTGACAGCCACGACCGACTGCGCA-3′). The SK-OV-3 cells were transduced by lentiviruses. The shRNA-coding lentiviruses were packed and harvested according to the manufacturer's protocol. Lentiviral transduction was performed according to the manufacturer's instructions. Briefly, 50,000 SK-OV-3 cells were plated in a 24-well plate on day 1. On day 2, the cells were primed with 8 μg/mL of hexadimethrine bromide, and viral particles were added to the culture at a multiplicity of infection (MOI) of 5. After 12 h of transduction, the virus-containing medium was replaced with fresh medium. Puromycin (2 μg/mL) selection started on the following day, and the surviving colonies were expanded for experiments.

### Xenograft animal model

Female athymic BALB/c nude mice were purchased from the Academy of Military Medical Science (Beijing, China). All mouse studies were approved by the Animal Ethics Committee of Tianjin Medical University Cancer Institute and Hospital. All protocols were approved and were under the supervision board of Tianjin Medical University Cancer Hospital and Institute. The experiments were carried out in accordance with the approved guidelines. All animals were 4-6 weeks of age at the time of injection. Each recipient animal received 1×10^7^ cells by intra-peritoneal injection. Cells were trypsinized, washed, re-suspended in Hanks' balanced salt solution (ThermoFisher Scientific, 14025092) and injected into the peritoneal cavities of the mice. SK-OV-3 cells were first incubated with the mouse anti-human CD47 mAb for 1 h at 20 μg/mL prior to xenograft. Control cells were incubated with the anti-HLA antibody. Additional antibody was administered once a week through intra-peritoneal injection at a dose of 100 μg. All animals were sacrificed a month after xenografting for necropsy.

### Sphere formation

The sphere assay was performed as previously reported [30]. Briefly, tumor cells isolated from ascites samples were incubated in stem cell conditions by resuspension in serum-free DMEM/F12 (ThermoFisher Scientific, 11330057) supplemented with 5 μg/mL insulin (Sigma-Aldrich, 91077C), 20 ng/mL human recombinant epidermal growth factor (EGF; PeproTech, 100-47), 10 ng/mL basic fibroblast growth factor (bFGF; PeproTech, 100-18B), 0.4% bovine serum albumin, 1 ng/mL hydrocortisone (Sigma-Aldrich, H0888), and 20 μg/mL gentamicin (Sigma-Aldrich, G1914) followed by culturing in Ultra Low Attachment plates (Corning, 07-200-601) and subsequent organization into spheres.

### Immunofluorescence analysis

Aldehyde dehydrogenase activity assays were performed using an ALDEFLUOR™ Kit (Stemcell Technologies, 01700). Briefly, ovarian cancer sphere cells were incubated in ALDEFLUOR™ assay buffer containing the ALDH substrate at 37°C for 45 min. To identify CD133-expressing cells and CD47-expressing cells, PE-conjugated antibody against CD133 (AC133, Miltenyi Biotec, 130-080-801) and FITC-conjugated antibody against CD47 (eBioscience, 110479) were used. Positive cells were quantified by ImagePro Plus software (Media Cybernetics, Inc., SilverSpring, MD). Images were acquired digitally using MagnaFire Software (Optronics).

### Cell viability

Cell viability was determined by MTT assays. Briefly, cells were seeded at an initial density of 5 × 10^4^ cells/mL in a 96-well plate for 24 h. Cells were then incubated at 37°C, 5% CO_2_ in a humidified incubator for 72 h. After incubation, 20 μL of MTT dye (5 mg/mL in DMEM) was added into each well. The insoluble formazan was collected, dissolved in dimethyl sulfoxide (DMSO) and measured at 570 nm with an ELISA reader (Bio-Rad).

### Cell proliferation assay

For proliferation assays, cells were seeded in 96-well plates at 4,000 cells/well. The proliferation rate was measured at 24, 48, 72, 96 and 120 h, using the MTT assay as described above. The analysis of proliferation rates in each individual experiment was repeated three times.

### Migration assay

For monolayer scratch wound-healing assay, cells were seeded in 6-well plates. To stop cell proliferation, the cells were maintained in DMEM containing 0.2% serum. At 100% confluence, a wound of 1 mm was made at the center of the well using a 200-μL-pipette tip. Cell migration after wounding for 0, 12 and 24 h was observed using a microscope. This experiment was repeated three times.

## SUPPLEMENTARY FIGURE AND TABLES


