# Supplementary material for: CD47 promotes ovarian cancer progression by inhibiting macrophage phagocytosis
Source: Oncotarget. 2017 Mar 24;8(24):39021–32. doi: 10.18632/oncotarget.16547 (PMC5503592; doi:10.18632/oncotarget.16547)
Supplement: Supplementary file 1 [file oncotarget-08-39021-s001.pdf]

CD47 promotes ovarian cancer progression by inhibiting macrophage phagocytosis

SUPPLEMENTARY MATERIALS

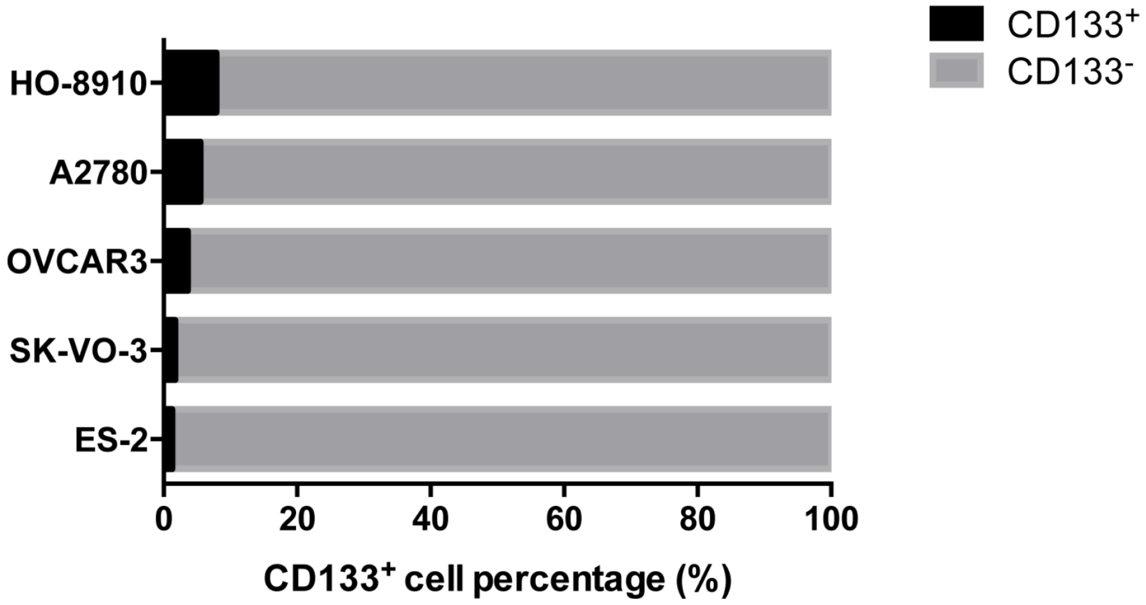

Supplementary Figure 1: Distribution of CD133$^+$ and CD133$^-$ subpopulations in ovarian cancer cell lines. Distribution of the CD133$^+$ and CD133$^-$ subpopulations were measured by flow-cytometry. CD133 was labelled with PE-conjugated anti human CD133 antibody (AC133, Miltenyi Biotec, 130-080-801). Flow cytometry data were acquired using a FACS C6 Flow Cytometer (Accuri Cytometer). At least 30,000 events were collected each analysis. All of the data were further analyzed by FlowJo software (Tree Star).

Supplementary Table 1: Tumor metastasis in mice xenografted with SK-OV-3 cells transfected with scramble shRNA or shCD47

Group	Numbers of tumor metastasis
Scramble (N=5)	55, 74, 70, 82, 97
shCD47 (N=5)	30, 42, 52, 61, 67

Supplementary Table 2: Tumor metastasis in anti-HLA antibody treated control mice and anti-CD47 mAb treated mice

Group	Numbers of tumor metastasis
Anti-HLA (N=5)	66, 71, 80, 88, 92
Anti-CD47 (N=5)	47, 52, 55, 72, 75
